# Simultaneous Dislocation of Radiocapitellar and Distal Radioulnar Joint

**DOI:** 10.1155/2013/106567

**Published:** 2013-10-01

**Authors:** Tomio Nishi, Noriyuki Suzuki, Takayuki Tani, Hiroshi Aonuma

**Affiliations:** Department of Orthopaedic Surgery, Ugo Municipal Hospital, 45 Otomichi Nishimonai, Ugomachi Ogachigunn, Akita, Japan

## Abstract

A 45-year-old male presented to the emergency room of our institution complaining of severe pain around the left elbow. While playing volleyball, he slipped down with his left arm hit between the floor and his body. He complaind of strong pain from left elbow to hand, and active motion of elbow and wrist joint was impossible. His forearm was held in supinated position. On X-ray examination, radius head was deviated to anterior lateral side, and distal end of radius was dislocated to dorsal side. Tenderness was prominent at the site of radial head and distal radioulnar joint. Surgical treatment was performed using triceps tendon strip. Good functional recovery was gained.

## 1. Background 

Dislocation or subluxation of radioulnar joint is usually seen in cases of trauma or rheumatoid arthritis. 

In children, anterior dislocation of radius head is often seen in case of Monteggia fracture [1]. Posterior dislocation of distal end of ulna is usually seen in case of rheumatoid arthritis or Galeazzi fracture. 

Simultaneous dislocation of radius head and distal end of ulna without other injuries are very few [2]. We experienced a case of simultaneous dislocation of proximal and distal ends of radius without fracture. Clinical course of surgical treatment of our case was very successful.

## 2. Present History 

A 45-year-old male presented to the emergency room of our institution complaining of severe pain around the left elbow on November 5, 2007. When he played volleyball and picked up ball, he slipped down with his left arm hit between the floor and his body. He complaind of strong pain from left elbow to hand, and active motion of elbow and wrist joint was impossible. 

## 3. Clinical Examination 

Forearm was held in supinated position.Deformity and swelling were prominent from his left elbow to wrist joint. Elbow joint was swollen and pressure pain was severe. Grip motion of hand was impossible due to pain.

Passive range of motion of elbow joint was flexion 90 degree/extension 0 degree, and passive range of motion of wrist joint was flexion −20 degree/extension 20 degree. Radius head was deviated to anterior lateral side, and distal end of radius was dislocated to palmer side.

Tenderness was prominent at the site of radial head and distal radioulnar joint.

## 4. Radiographic Findings 

**Figure 1:**
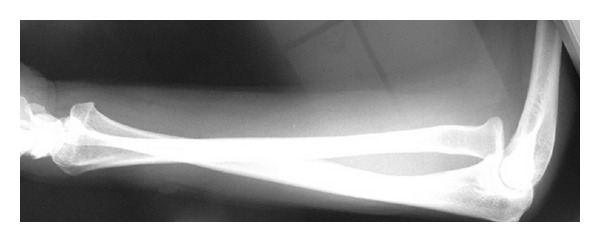
Radiographic findings at the first medical examination. From lateral view, radius head was dislocated anteriorly and distal end of radius was dislocated posteriorly. Radius and ulna were found to have the shape of letter X.

From lateral view, radius head was dislocated anteriorly and distal end of radius was dislocated posteriorly. Radius and ulna were found to have the shape of letter X (Figure 1) [2–6].

## 5. Surgical Treatment

**Figure 2:**
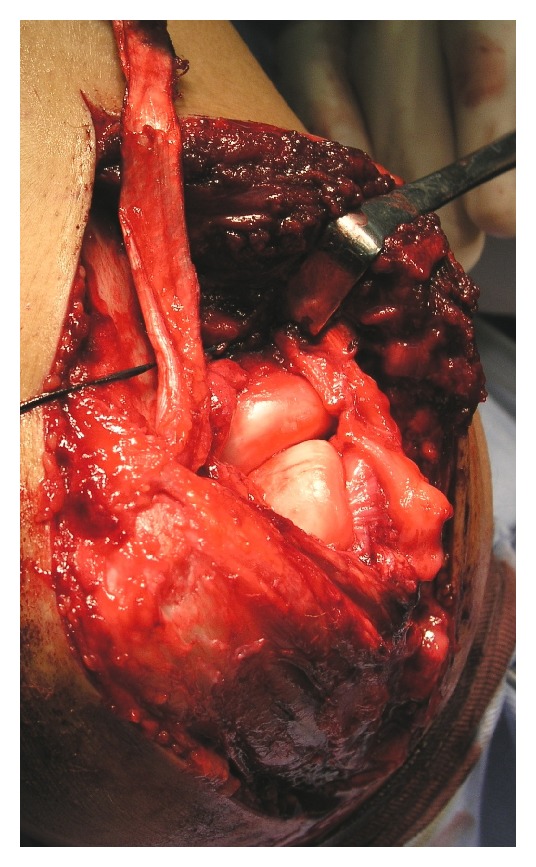
Fascia flap of triceps of 7 mm width and 100 mm long was made, which was passed from posterior to anterior around the head of radius.

**Figure 3:**
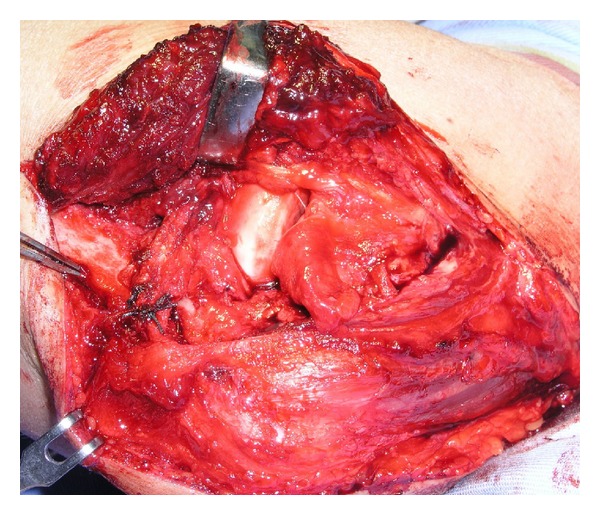
Fascia flap of triceps was passed around radius head, tied on the base of flap by nonabsorbable suture. The arm was then immobilized in full extension and supinated.

On the third day of injury, manual reduction was tried under general anesthesia, but reduction was impossible. So, we shifted to surgical treatment. By using posterior-lateral approach, elbow joint was opened. Joint was filled with dark reddish brown color fluid. Radius head was dislocated forward and repositioned by internal rotation. But proximal radioulnar joint was very unstable and easily dislocated forward. We decided to cut radial collateral ligament in z-shape to visualize the annular ligament. Direct repair of broken thin annular ligament was impossible. We decided to make fascia flap of triceps 7 mm width and 100 mm long, which was passed from posterior to anterior around the head of radius and tied on the base of flap with nonabsorbable suture. The arm was then immobilized in full extension and full supination (Figures 2 and 3) [1, 7, 8].

**Figure 4:**
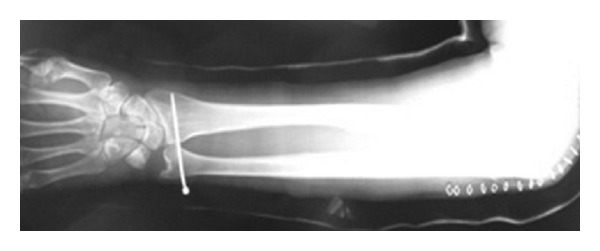
Distal radioulnar joint was manually repositioned. 1.5 mm Kirchner wire was passed through the joint.

 Distal radioulnar joint was manually repositioned. 1.5 mm Kirchner wire was passed through the joint (Figure 4). After the surgery, a long arm posterior splint was used. Active range of motion exercise of elbow joint was started three weeks later. Rotation exercise of forearm was started five weeks later. 

## 6. Postoperative Course

**Figure 5:**
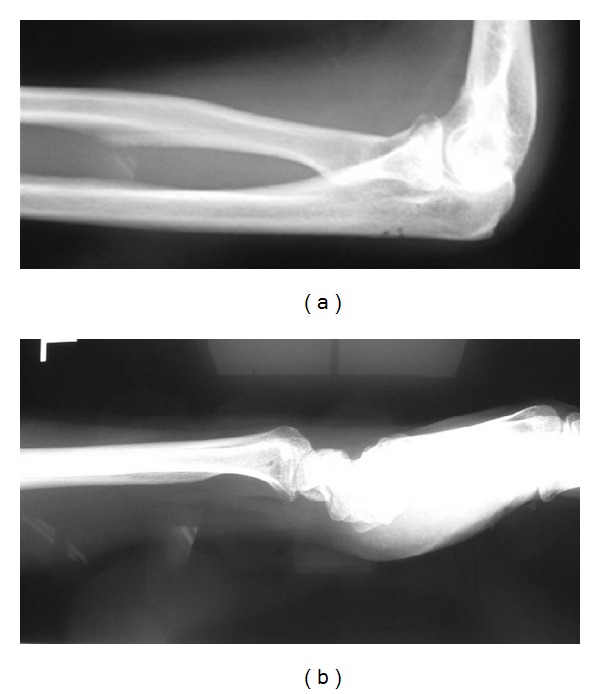
One year after the operation, proximal and distal radioulnar joint was in normal position.

**Figure 6:**
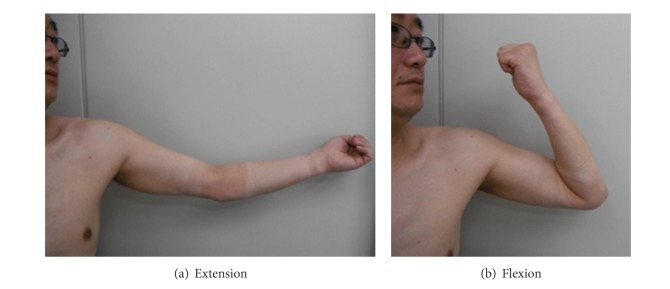
One year after the operation: (a) extension; (b) flexion.

**Figure 7:**
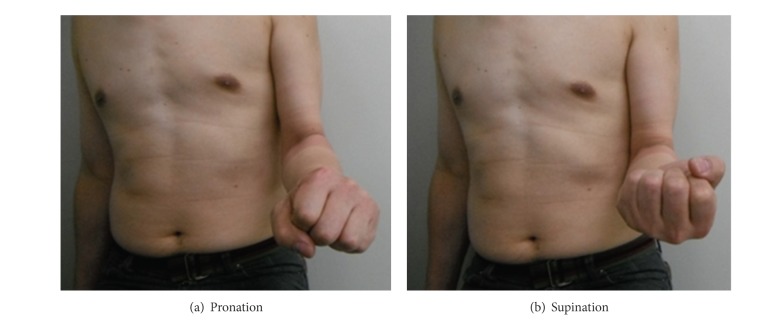
One year after the operation: (a) pronation; (b) supination.

On October 11, 2011, forty-nine months after the injury, patient's complaint was no pain or numbness. Active range of motion of elbow joint was flexion 140 degree/extension −20 degree (Figure 6), and rotation of forearm was supination 90 degree/pronation 70 degree (Figure 7). Active range of motion of wrist joint was flexion 70 degree/extension 70 degree. Radiographic finding of proximal and distal radioulnar joint was in normal position (Figures 5(a) and 5(b)). 

## 7. Discussion

Anterior dislocation of radius head is mostly seen in Monteggia fracture. Posterior dislocation of distal end of ulna is seen in case of rheumatoid arthritis or Galeazzi fracture. Simultaneous dislocation of radius head and distal end of ulna without other injury are very few.

Spicer et al. (2002) [5] and Potter and Wang (2012) [6] described persistent radiocapitellar subluxation after closed reduction of an elbow dislocation, secondary to irreducible volar subluxation of the DRUJ. 

Leung et al. (2005) [3, 4] presented the first case of simultaneous dislocation of proximal and distal radioulnar joint without fracture.

Verettas (2008) [2] presented the second case of simultaneous dislocation of radial head and distal radioulnar joint without any injury.

Tosun et al. (2008) [9] presented a case of isolated interosseous membrane disruption without any fracture. In this case, dislocation of both radial head and distal radioulnar joint was seen.

In case of simultaneous dislocation of the radial head and distal radioulnar joint, Leung et al. [4] suggested that inter-osseous membrane may play a role as a pivot between radius and ulna.

Based on cadaveric studies, it is believed that even in an isolated dislocation of one of the radioulnar joints, subluxation or sprain of other joint is present. 

At proximal radioulnar joint, annular ligament plays the most important role in rotation and stability of ulna head. In our case, annular ligament was completely broken, and direct repair of this ligament was impossible.

Thompson and Lipscomb [10] used fascia lata graft to reconstruct annular ligament for recurrent radial head subluxation. But in our case, energy of trauma was intense, soft tissue damage around radius head was remarkable, and we reconstructed new annular ligament of radius by using triceps tendon flap (modified Bell-Tawse procedure [11]). Reconstructed new annular ligament had brought enough stability to humeroradial joint.

Ulna distal end was manually brought into normal position. Distal radioulnar joint was transfixed with Kirschner wire for four weeks.
